# Ferritin Nanocage: A Versatile Nanocarrier Utilized in the Field of Food, Nutrition, and Medicine

**DOI:** 10.3390/nano10091894

**Published:** 2020-09-22

**Authors:** Chenxi Zhang, Xiaorong Zhang, Guanghua Zhao

**Affiliations:** College of Food Science and Nutritional Engineering, Beijing Key Laboratory of Functional Food from Plant Resources, China Agricultural University, Beijing 100083, China; zhangchenxi9556@163.com (C.Z.); zhangxiaorong1204@163.com (X.Z.)

**Keywords:** ferritin nanocage, nanocarrier, encapsulation, delivery

## Abstract

Compared with other nanocarriers such as liposomes, mesoporous silica, and cyclodextrin, ferritin as a typical protein nanocage has received considerable attention in the field of food, nutrition, and medicine owing to its inherent cavity size, excellent water solubility, and biocompatibility. Additionally, ferritin nanocage also serves as a versatile bio-template for the synthesis of a variety of nanoparticles. Recently, scientists have explored the ferritin nanocage structure for encapsulation and delivery of guest molecules such as nutrients, bioactive molecules, anticancer drugs, and mineral metal ions by taking advantage of its unique reversible disassembly and reassembly property and biomineralization. In this review, we mainly focus on the preparation and structure of ferritin-based nanocarriers, and regulation of their self-assembly. Moreover, the recent advances of their applications in food nutrient delivery and medical diagnostics are highlighted. Finally, the main challenges and future development in ferritin-directed nanoparticles’ synthesis and multifunctional applications are discussed.

## 1. Introduction

With the development of nanotechnology and material science, nanosized materials with high stability and efficient cell permeability have been widely used in encapsulation and delivery system, including liposomes, micelles, block copolymers, carbon nanotubes, dendrimers, oligosaccharides, and protein cages [1]. Among myriad nanocarriers, protein nanocages have received more and more attention owing to their inherent cavity size, excellent biocompatibility, and high cellular uptake efficiency with minimal toxicity [2,3]. The most recent development in designing protein nanocarrier has been focused on a ferritin delivery platform because of their unique features, such as self-assembly and biomineralization. Ferritin is a specific class of iron storage and detoxification proteins with different patterns that can be found in humans, plants, and other living organisms, and it plays a pivotal role in maintaining iron homeostasis in a soluble, nontoxic, and bioavailable form [4,5]. One ferritin molecule can accumulate up to ~4500 iron atoms within its inner cavity, and such a form is named as holoferritin. However, upon removal of iron from the cavity by reduction of Fe (III) and subsequent chelation of Fe (II) [6,7], holoferritin can be easily converted into its apo form, which is named as apoferritin. Interestingly, apoferritin is very stable, even when heating to 80 °C for 10 min, and it can tolerate harsh chemicals such as urea and guanidinium chloride [8,9]. More importantly, the empty inner cavity of apoferritin has been explored as a versatile nanoreactor for synthesizing water-soluble, heat-resistant, and size-restricted nanoparticles [10]. Compared with other nanocarriers, ferritin has several advantages: (1) it can be produced economically in *Escherichia coli* and can be purified easily by exploiting its heat-proof property [11]; (2) it exists naturally in the human body and is composed of nontoxic elements that thus would not elicit strong nonself antibody and/or T cell immune responses [12]; (3) ferritin cage is disassociated into subunits at extremely acid/alkaline pH (≤2.0 or ≥11.0) and resulting subunits can reconstitute into a cage-like structure at neutral conditions, and by taking advantage of this feature, guest molecules can be easily and effectively encapsulated into the ferritin cavity [13,14]; (4) ferritin has a ferroxidase site or nucleation site, which can bind iron ions and other metal ions [15]; and (5) it can be easily functionally modified by genetic and/or chemical coupling [16]. So far, ferritin has been utilized to encapsulate multifarious food bioactive nutrients such as anthocyanin, β-carotene, curcumin, rutin, and epigallocatechin gallate to improve their water-solubility, stability, and cellular uptake efficiency [14,17,18,19,20]. In addition, therapeutic drugs and contrast agents are accommodated into the ferritin cavity to realize target delivery, cell imaging, and tumor therapy [21,22].

This review presents an overview of recent advances in ferritin-based nanocarriers. First, the structural characterization, self-assembly, and iron oxidative deposition properties of ferritin will be summarized in order to familiarize the reader with them. Then, we mainly focus on the preparation of the ferritin-hybrid nanoparticles by taking advantage of protein structural characteristics, regulation of physical conditions, addition of chemicals, and genetic modification strategies. Moreover, the applications of the resulting hybrid materials and nanostructures in food nutrient delivery and medical diagnostic will be highlighted. Last, but not least, we discussed the current challenges and future efforts in developing ferritin-based nanoparticles for multifunctional applications.

## 2. Structure and Property of Ferritin

### 2.1. General Aspects of Ferritin Structure

**Figure 1:**
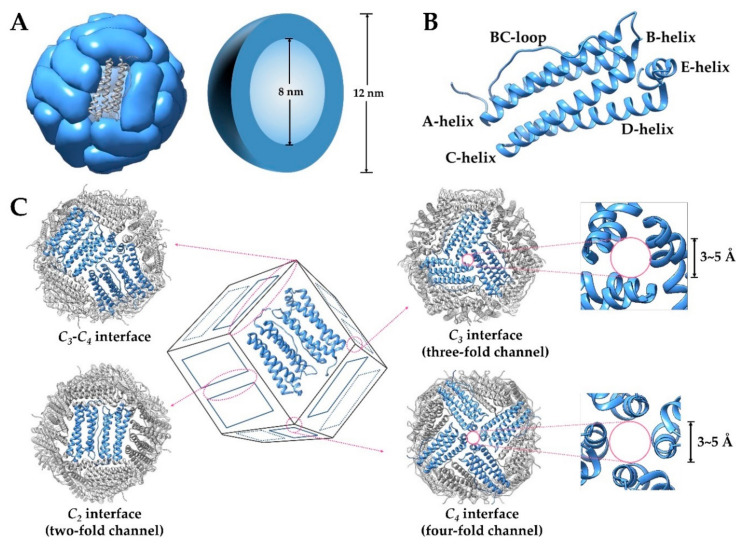
(**A**) Structure of ferritin and its outer/inter diameter. (**B**) Graphic representation of ferritin subunit structure. (**C**) The inter-subunit interfaces of ferritin and the pore size at two different interfaces.

Ferritin is a ubiquitous and well-characterized iron storage and detoxification protein, whose structure is highly conserved among plants, animals, and bacteria [6]. A typical ferritin is composed of 24 identical or similar subunits (see after), which self-assemble to form a hollow protein cage with an outer diameter of 12~13 nm, an interior cavity diameter of 7~8 nm, and a thickness of 2~2.5 nm (Figure 1A). Every two subunits of ferritin form a group in antiparallel, and then these twelve pairs of subunits form an approximately regular dodecahedron, with 4-3-2 axial symmetry (Figure 1C). Each subunit of ferritin is around cylindrical (about 5 nm in length and 2.5 nm in width), consisting of a four-α-helix bundle containing two antiparallel helix pairs (A, B and C, D) and the fifth shorter α helix (E) at the C-terminal. The B and C helices are connected by a long nonhelical stretch of 18 residues (the BC-loop), and the E helix is located at the end of the four-α-helix bundle and forms an angle of 60° with it (Figure 1B). The N-terminal, BC loop, A-helix, and C-helix of subunits form the outer surface of ferritin, while the B-helix and D-helix face the inner surface [6,23,24]. Under neutral conditions, the inner surface of ferritin has a high negative charge density because acidic residues such as Glu and Asp are mainly distributed here, while the net charge on the exterior surface is close to zero or slightly positive. One ferritin molecule has eight triple axis channels and six quadruple axis channels with pore sizes between approximately 3 and 5 Å (Figure 1C), which connect the inner cavity to the external environment [25].

### 2.2. Animal Ferritin

**Figure 2:**
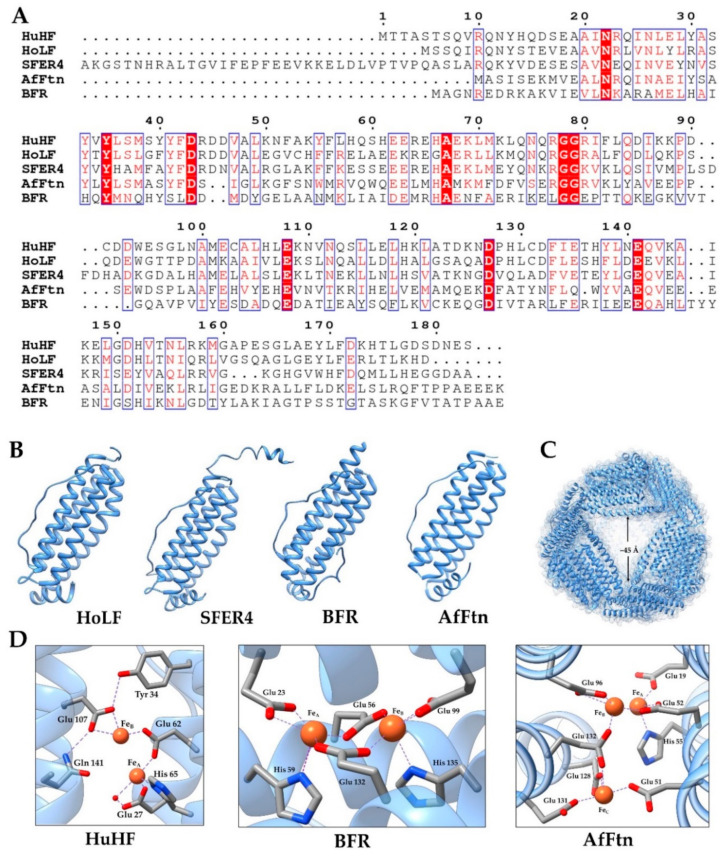
(**A**) Amino acid sequences of human H chain ferritin (HuHF), horse L-chain ferritin (HoLF), soybean ferritin 4 (SFER4), haem-containing bacterial ferritin (BFR), and *Archaeoglobus fulgidus* ferritin (AfFtn) [33]. (**B**) The subunit structure of HoLF, PDB 2v2i [34]; SFER4, PDB 3a68 [24]; BFR, PDB 1nfv [35]; and AfFtn, PDB 1s3q [36]. (**C**) The structure of AfFtn viewed down one of four large pores at a three-fold noncrystallographic symmetry axis. (**D**) The ferroxidase center of HuHF, BFR, and AfFtn.

For mammals, ferritin is usually composed of two types of subunits, H (heavy chain, molecular weight about 21 kDa) and L (light chain, molecular weight about 19.5 kDa), and the amino acid sequences of the two subunits have 55% similarity (Figure 2A). The H-type subunit contains a dinuclear ferroxidase center composed of A and B iron-binding sites of conserved amino acid ligands Glu27, Tyr34, Glu62, His65, Glu107, and Gln141, mainly responsible for the rapid oxidation of ferrous ions (Figure 2D). One ferrous oxidation center can combine two Fe^2+^ ions at the same time [6,15,23,26]. However, the L-type subunit lacks the ferroxidase center, but it contains a putative nucleation site that is responsible for the slow oxidation of ferrous ions and the formation of a mineral core [27]. The proportion of subunits varies between different organisms and between different tissues within an organism, reflecting the different functions, fast iron metabolism, or long-term iron storage required by different tissues [16,28]. In addition, for lower vertebrates, such as bullfrogs, fish, and tegillarca granosa, there is also an M (medium) subunit or H-like subunit containing both the ferroxidase center and the nucleation site in the ferritin of erythrocyte, and the three subunits can form heterozygous ferritin [25,29]. Differently, the mitochondria ferritin (MtF) found in human, mouse, and drosophila is a homopolymer with 24 identical subunits. The subunits of MtF are synthesized as 30 kDa precursor proteins that is targeted to mitochondria. After intramitochondrial deletion of the N-terminal leader sequence, the 22 kDa MtF subunits assemble into ferritin shells with ferroxidase activity [30]. The homology of human mitochondrial ferritin subunit and human H chain subunit is about 79% [31]. In animal ferritin, the three-fold channel is hydrophilic and the four-fold channel is hydrophobic. Therefore, the three-fold channels have been considered as a main entry or exit pore, and a site for Fe (II) oxidation [32].

### 2.3. Phytoferritin

Plant ferritin has many special characteristics compared with animal ferritin. Firstly, phytoferritin is observed in plastids, and the gene expression is regulated at the transcriptional level, while animal ferritin is mainly present in the cytoplasm of cell, and the expression is strictly controlled by the interaction of iron response elements and iron regulatory proteins, which belongs to the transcription level [37,38]. Secondly, the subunits that constitute plant ferritin are all H-type, and each subunit contains a ferrous oxidation center, and its amino acid composition is about 40% similar to animal H subunit (Figure 2A). Phytoferritin isolated from pea, soybean, black bean, corn, alfalfa, and arabidopsis are composed of H-1 (26.5 kDa) and H-2 (28.0 kDa), with 80% amino acid sequence homology [7,39,40]. Thirdly, the extension peptide (EP) is an important component unique to the N-terminus of plant ferritin subunits. The crystal structure of recombinant soybean seed ferritin indicates that EP (~30 amino acid residues) is located on the outside surface of the protein and stabilizes the entire oligomeric conformation of plant ferritin through interaction with adjacent subunits on the shell surface [24]. On the one hand, as the second ferroxidase center, EP is responsible for mineralization of the iron core during iron oxidative deposition in phytoferritin (>48 irons/shell) [41]. On the other hand, the EP exhibits a serine protease-like activity, and is also responsible for auto-degradation of ferritin [42]. Fourthly, each quadruple channel of plant ferritin is lined with eight histidine, so it is more hydrophilic than animal ferritin, which is mainly rich in leucine and more hydrophobic groups [40]. Recent studies of our group reveal that both three-fold and four-fold hydrophilic channels are necessary for iron diffusion into ferritin [43]. Last, because all E-helices from 12 H-1 subunits have been deleted after assembly with H-2 into protein shell, the four-fold channels of ferritin from mature soybean seeds appear to have larger aperture [40].

### 2.4. Bacterial Ferritin

Generally, there were two types of ferritin found in prokaryotes thus far (DNA binding proteins from starved cell are not discussed in this article), the archetypal ferritins of the H type, which are also found in eukaryotes (FTNs), and the haem-containing bacterioferritins (BFRs) (Figure 2B) [44]. The majority of FTNs have the canonical octahedral (4-3-2) symmetry, and consist of 24 subunits similar to mammalian H-chain ferritins [45]. However, the hyperthermophilic *Archaeoglobus fulgidus* ferritin (AfFtn) assembles in a unique manner into a roughly spherical shell with tetrahedral (2-3) symmetry, and contains four large pores with a diameter of about 45 Å (Figure 2C) [36]. In addition, the AfFtn and *Thermotoga maritima* ferritin (TmFtn) dissociate from 24-mer to dimers at low ionic strengths, and by addition of specific metal ions such as Fe (II), the dimer will be reassembled as canonical 24-mer [36,46]. Similar to the FTNs, BFRs are made up of 24 H-type subunits, most of which are homopolymers with 4-3-2 symmetrical arrangement. Differently, BFRs contain 12 hemes, which are located at the interface of two-fold symmetry related subunits and bind to the protein through two methionine residues. Studies show that the hemes in *Escherichia coli* BFR and *Pseudomonas aeruginosa* BFR have a significant effect on iron release instead of iron uptake [47]. Similar to the ferroxidase centers of recombinant human H chain ferritin (HuHF), both FTNs and BFRs have a glutamate bridging both metal centers and each iron atom has an axial glutamate ligand. Otherwise, the FTNs in *Escherichia coli*, *Archaeoglobus fulgidus,* and *Pyrococcus furiosus* have a third iron site located about 0.6–0.7 nm away from sites A and B, while BFRs have a second bridging Glu (Figure 2D) [23,45].

### 2.5. Non-Native Ferritin

**Figure 3:**
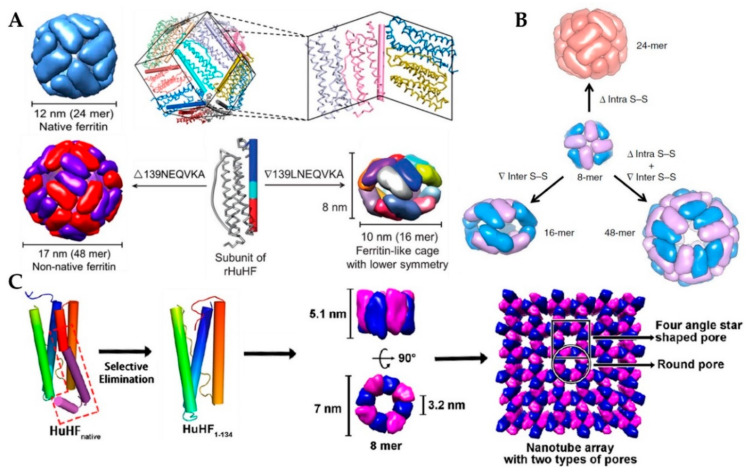
(**A**) Schematic representation of conversion of native human ferritin into its 48-mer and 16-mer analogues (Reproduced with permission from [48,49], *ACS Nano* and *Angew. Chem. Int. Ed.*, 2016, respectively.). (**B**) Schematic representation of the conversions from 8-mer bowl-like proteins to protein nanocages with different sizes and geometries (24-mer, 16-mer, and 48-mer) (Reproduced with permission from [50], *Nat. Commun.,* 2019). (**C**) Conversion from 24-mer protein nanocage into nanorings with *D_4_* symmetry (Reproduced with permission from [51], *J. Am. Chem. Soc.*, 2018).

Because the size and shape of native ferritin are limited to meet all specific application requirements, scientists have tried to construct several ferritin-derived proteins through genetic engineering. Therein, by reengineering the key subunit interfaces of native 24-mer protein cage, our research group recently has fabricated its 48-mer non-native analogue and 16-mer lenticular nanocage through deletion of ^139^NEQVKA^144^ or insertion of ^139^LNEQVKA^145^ motif in the middle of the D-helix, respectively, as shown in Figure 3A [48,49]. X-ray crystal diffraction results show that this 48-mer nanocage was composed of two types of subunits with the same amino acid sequence, but different configurations, called H_α_ and H_β_. Despite the same octahedra symmetry, its two-fold and three-fold undergo great changes: (1) the *C_2_* interface consists of C- and D-helix and the CD turn of two H_α_ subunits; (2) the three-fold channel involves three pairs of H_α_ (AB turn, BC loop, and C-helix) and H_β_ (the N-terminal, B-helix, and BC loop) subunits contributed from three adjacent tetramers, producing large pores with a size of ~25 Å. The outer diameter has also changed from 12 nm of natural protein to 17 nm, but in the solution, it will be converted into an 8-mer bowl-like oligomer with a diameter of about 10 nm. Different from this 48-mer and native 24-mer protein cage, a novel 16-mer nanocage is an approximately oblate spheroid with a long axis of 10 nm and a short axis of 8 nm. The crystal structure shows 16-mer is devoid of the three-fold interactions, but forms *pseudo*-*D_2_* and -*D_4_* axes. This 16-mer nanocage contains two similar 8-mer moieties, which were bridged mainly relying on the hydrophobic interactions along *pseudo*-*D_2_* axes. The *pseudo*-*D_2_* interface consists of the C-terminal end of helix A, the N-terminal end of helix B, as well as N-terminal end of helix C. In the crystal structure, there is a large “window” (four in the whole molecule) in the perpendicular directions of the 16-mer nanocage, with an aperture of about 65 nm, which actually obscured by disordered C-terminal tails of two monomers [48,49]. Subsequently, using the above-mentioned 8-mer non-native protein as the building block, a set of discrete protein nanocages with different sizes and geometries (24-mer, 16-mer, and 48-mer) was constructed through deletion of one inherent intra-subunit S–S bond formed within one subunit, insertion of inter-subunit S–S bonds at the protein interface, and deletion of the intra-subunit S–S bonds along with insertion of the inter-subunit S–S bonds, respectively (Figure 3B) [50].

More interestingly, after removal of 49 residues from the C-terminal of each ferritin subunit, the native 24-mer protein cage converted into 8-mer protein nanorings with *D_4_* symmetry (Figure 3C). The inner wall of the nanorings is composed of only helix B, while its outer wall comprises helix A, helix C, and BC loops. The height of this nanoring is 5.1 nm, and its inner diameter is 3.2 nm and outer diameter is 7 nm. It should be noted that the nanoring is only stable in the pH range of 8.0–9.0 at temperatures from 25 to 35 °C. In the crystal, such nanorings connect with each other in a repeating head-to-tail pattern to form nanotubes, and adjacent nanotubes are staggered relative to one another to create three-dimensional porous protein assemblies [51].

## 3. Preparation of Ferritin-Hybrid Nanoparticles

**Figure 4:**
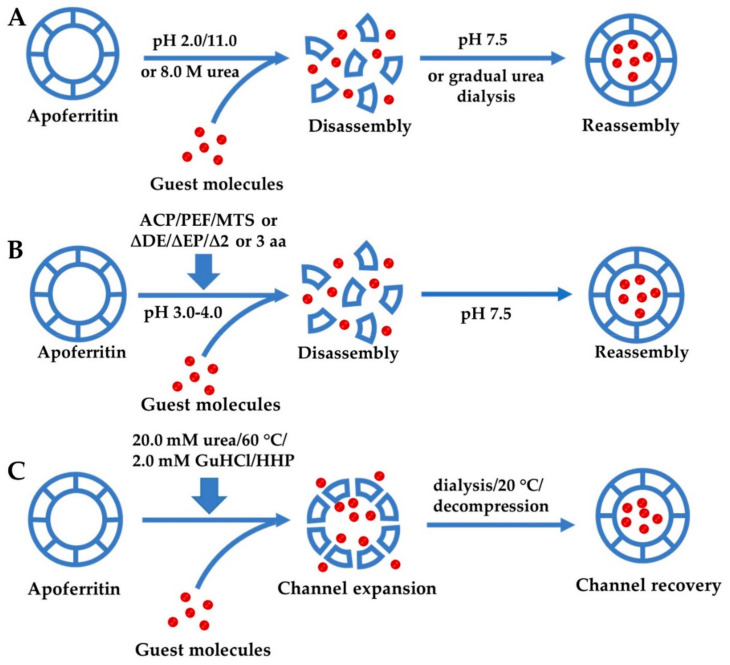
Schematics for the fabrication process of guest molecules encapsulated within the ferritin cavity by different mechanisms: (**A**) self-assembly of ferritin controlled by different chemicals; (**B**) regulation of ferritin self-assembly by physical methods or genetic modification under relatively mild conditions; (**C**) expansion of ferritin channels by different methods. ACP, atmospheric cold plasma; PEF, pulsed electric field; MTS, manothermosonication; EP, extension peptide; HHP, high hydrostatic pressure.

The structure of ferritin determines that it has a variety of functions, such as high stability and cell selectivity. Among them, reversible self-assembly and channel sensitivity have attracted great interest in the field of nanocarriers. In recent years, researchers have controlled their self-assembly and channel size through different factors, and developed a variety of methods to prepare ferritin-hybrid nanoparticles. The schematics for the fabrication of guest molecules encapsulated within ferritin cavity are shown in Figure 4 and their features are summarized in Table 1.

### 3.1. Reversible Self-Assembly Property of Ferritin

Self-assembly is necessary for generating large, complex supramolecular structures with multiple biological functions, such as microtubules, bacterial flagella, virus capsids, and ferritins [52]. Gerl and Jaenicke monitored the folding and assembly of heteropolymeric horse spleen apoferritin (HSF) via chemical cross-linking and subsequent spectroscopic analysis, and they thought that the overall kinetic mechanism may be described by the following scheme [53]:

24 m_i_ → 24 M_1_ ⇆ 8 M_1_ + 8 M_2_ ⇆ 8 M_3_ ⇆ 4 M_6_ ⇆ 2 M_12_ → M_24_

where the subscript denotes the number of structured subunits, and mi is the unfolded monomers. Whereafter, time-resolved small-angle X-ray scattering (TR-SAXS) was used to measure the assembly reaction of homopolymeric *Escherichia coli* ferritin A (EcFtnA). Different from heteropolymeric HSF, monomers and trimers are unlikely to be intermediates during the assembly of homopolymeric ferritin. The time-dependent change in the TR-SAXS profile was roughly explained by a modified model:

12 M_2_ ⇆ 6 M_4_ ⇆ 4 M_6_ ⇆ 2 M_12_ → M_24_

Furthermore, researchers also shed light on the role of electrostatic interactions on the assembly by the combination of TR-SAXS with site-directed mutagenesis [52,54]. However, more details about the mechanism of ferritin self-assembly need to be further elucidated. Up to now, different research groups have proposed and implemented a variety of approaches to control protein self-assembly property and applied them to the preparation of guest molecule-loaded ferritin nanocarrier. In this review, we divide them into three categories, including strategies based on chemical substance addition, physical condition regulation, and genetic modification.

#### 3.1.1. Self-Assembly of Ferritin Controlled by Different Chemicals

As mentioned previously, ferritin can be decomposed into individual subunits under extremely acidic/alkaline conditions (pH ≤ 2.0 or pH ≥ 11.0). The subunits maintain native-like secondary and tertiary structures and can reassemble into a 24-mer spherical structure when the pH returns to neutral, which provides an efficient way to encapsulate cargo molecules that are unable to diffuse into the cavity of the intact protein shell through the pores. On the basis of this reversible self-assembly property, plenty of nutrients, drugs, and makers have been accommodated into the ferritin nanocage [14,17,18,22,55]. As shown in the schematic (Figure 4), first, the ferritin solution pH was adjusted to ~2.0/11.0 by adding the appropriate volume of 1.0 M HCl/NaOH to disassemble ferritin into subunits. Second, the stock solution of guest molecular (nutrients, drugs, or makers) was added to such ferritin disassociated solution slowly. Third, the pH value was adjusted back to 7.5 using 1.0 M HCl/NaOH and the resultant mixture was stirred at 4 °C for 2 h to promote the assembly of the protein. Therewith, the resulting solution was dialyzed against buffer or ultra-filtrated or centrifuged to remove free guest molecules. Finally, the ferritin-hybrid nanoparticles solution was stored at 4 °C for later use [17,18]. Through this process, guest molecular could be trapped and retained inside the apoferritin shell because its size is larger than the pore size of the protein channels (3–5 Å) [24]. So far, a variety of bioactive molecules such as anthocyanin [14], rutin [56], and β-carotene [17] have been accommodated within the cage of ferritin using the disassembly and reassembly process. Depending on the guest molecule, the encapsulation rate can reach 15–32%. Moreover, the resulting nanocomposites can realize solubilization, stabilization, delivery, and controlled-release of the nutrients and drugs [18,21,57]. However, the pH-induced apoferritin disassembly and reassembly processes were not fully reversible and would form hole defects on the hollow spherical structure [58]. Moreover, only about 60% of disassociated protein molecules could reassemble into a protein nanocage after pH was adjusted back to neutral [59].

Particularly, AfFtn and TmFtn show salt-sensitive assembly features, that is, they exist as dimers in low-salt environments and reconstitute into a cage-like structure in high-salt environments [60]. The reversible self-assembly property of different apoferritin provides a convenient route to encapsulate guest molecules into its cavity. Besides, AfFtn is unique in its tetrahedral quaternary structure and the presence of four large triangular pores with a diameter of about 45 Å (~2500 Å^2^ pore area), which provide the possibility for large cargos to enter its cavity [36]. Coupled with the negatively charged interior of AfFtn, a highly positively charged green fluorescent protein (GFP) variant (+36GFP, pI 10.4), several enzymes (human carbonic anhydrase II and the artificial (retro-)aldolase RA95.5-8F and Kemp eliminase HG3.17) genetically fused to +36GFP [61], and cytochrome C (pI = 10.8) [62] can be encapsulated within the ferritin cavity by simply mixing with protein solution containing NaCl/MgCl_2_. Through quantitative analysis, each protein cage can accommodate five GFP molecules or two to three GFP–enzyme complex molecules or 1.4 cytochrome C. However, it may not be suitable for the encapsulation of multimeric or highly negatively charged enzymes owing to the relatively small size of the cavity and/or reduced electrostatic attraction [61]. Moreover, the large pores may make cargo vulnerable to attack from degrading agents or easily diffuse out of the cavity. As another ferritin that showed salt-induced assembly, TmFtn displays octahedral symmetry and has a closed shell [60]. As TmFtn carries an overall negative charge on its internal lumen similar to AfFtn, it can also encapsulate positively charged cargos (+36GFP or lysozyme) by simple mixing of TmFtn along with these cargos in the presence of 50 mM MgCl_2_. Quantitative analysis found an average of ~4 GFPs or ~5 lysozyme molecules loaded inside each TmFtn cage under the optimized conditions. In addition, the disassembly of TmFtn cage and cargo release can be triggered by incubating overnight with 100 mM ethylene diamine tetraacetic acid (EDTA), but such salt-regulated self-assembly is only found with these two archaeal proteins.

Not only the acid/base reagent, but also the high concentration of chaotropic agent such as urea and guanidine hydrochloride (GuHCl) could contribute to the disassembly of ferritin into subunits by destroying the non-covalent forces (e.g., hydrogen bonds and hydrophobic effects) [63,64]. Liang et al. has prepared HuHF-hybrid nanoparticles through disassembling HuHF in 8 M urea in the presence of doxorubicin (Dox), followed by a reassembling process with a series of stepwise gradients of urea from 8 M to 0 M in buffer [21]. Because reassembly needs some time to restore its initial conformation, DOX is added to the dialysate to avoid leakage of the encapsulated molecules during the recovery process. As the pK_a_ of Dox is 8.2, it can easily bind to the negatively charged inner surface of ferritin through electrostatic interactions in the neutral buffer. Size-exclusion chromatography showed a single absorbance peak in both 280 nm (HuHF) and 485 nm (Dox) UV traces, which confirmed the successful loading of Dox into HuHF nanocages. According to the concentration calculation, each protein cage contains about 33 molecules of doxorubicin. Moreover, Dox loading has no effect on the structure of HuHF, indicating that the protein will refold into its native state upon loading. For this method, protein precipitation is still unavoidable during the dialysis process.

#### 3.1.2. Regulation of Ferritin Self-Assembly by Physical Methods

Taking the above facts into account, it is necessary to establish a new approach to encapsulate guest molecules within ferritin nanocages, which can maintain not only the bioactivity of targeted small molecules, but also the integrity of the ferritin shell-like structure. Atmospheric cold plasma (ACP) is a source of reactive oxygen species, which refers to the non-equilibrium plasma produced at the temperature and pressure close to the environment. It has great potential in surface hydrophobicity enhancement, surface regulation, and enzyme/microorganism inactivation. After evaluating the structure and property of ACP-treated ferritin (ACPF), it was found that the ACP treatment could affect the microenvironment of tryptophan around the ferritin channels, reduce the α-helix/β-sheet contents, lead to a decrease in aromatic surface hydrophobicity and thermal stability, and influence the access and release of iron. More importantly, ACPF can dissociate into subunits at pH 4.0, which is a more benign condition than that of the untreated ferritin. Thus, the bioactive molecules could be added into the protein solution at pH 4.0 and encapsulated in the cage when the pH returns to the neutral range. By this way, curcumin was successfully encapsulated within the ACPF cage and the encapsulation ratio of 12.7% was obtained [65]. As another non-thermal technology, pulsed electric field (PEF) has similar effects on the structure and features of ferritin as ACP. PEF-modified ferritin could disassemble at pH 3.6 and encapsulate rutin into its cavity with the encapsulation ratio of 13.7% [66]. Manothermosonication (MTS) is a new ultrasonic treatment method, which has the effect of generating bubble explosion and improving acoustic cavitation activity. It has been reported that MTS can modify the structure and properties of soy protein and improve its physical and chemical properties, including solubility, free sulfhydryl groups, surface hydrophobicity, and antioxidant activity [67]. A recent study has proved that MTS treatment can decrease the ferritin stability, thus affecting its iron oxidative deposition and iron release activity. Accordingly, the MTS-treated apoferritin could encapsulate epigallocatechin gallate (EGCG) by a relatively benign pH conversion routine (pH 3.0/6.8) and the encapsulation ratio can reach ~25% [68]. These studies expand the application of these physical technologies in the encapsulation field of cargo molecules by ferritin.

#### 3.1.3. Genetic Modification for Controlling Ferritin Self-Assembly

In addition to the above approach, genetic modification is also a potential strategy for preparing ferritin-hybrid nanoparticles. As cage-like protein, ferritin has three distinct interfaces: the interior surface, the exterior surface, and the interface between subunits. Among them, the interface between subunits is generally manipulated by a variety of non-covalent interactions, such as hydrogen bonding, electrostatic association, hydrophobic effects, and van der Waals forces. Appropriate modification of these interfaces may result in novel or analogous nanostructures with special properties. As the *C_4_* interface is only composed of E-helix and DE-turn contributed by four subunits, it is easier to modify by genetic methods than other interfaces. After deleting the last 23 amino acids involved in the formation of the DE-turn and E-helix, recently, our research group has prepared a new protein named rHuHF-ΔDE [69]. The deletion has almost no influence on the shell-like structure, thermal stability, and iron oxidation deposition of ferritin. Surprisingly, this engineered ferritin can be dissociated into subunits at pH 4.0 and reassemble at neutral, which is different from HuHF, which disassociated at pH 2.0. By utilizing its reversible disassembling and reassembling properties under benign pH conditions, bioactive compounds and drugs that are susceptible to pH could be encapsulated into its cavity without degradation or isomerization. However, large pores up to 18 Å appear at each fourfold channel of rHuHF-ΔDE, thus small molecules encapsulated within the cavity of ferritin might leak out. Analogously, Ahn B. et al. eliminated the carboxyl terminal short E-helix of rHuHF (F160) and fabricated a new nicked ferritin by mixing the rHuHF and F160 with ratios of 1:1–2:1; the results show that the nicked-HuHF starts to be disassembled at pH ~4.0 [70]. Similarly, the phytoferritin could also disassemble at pH 4.0 and reassemble at pH 6.7 upon deleting its EP domain [71].

To avoid large pores caused by deleting E-helix, we have engineered two new mutants named rHuHFΔ2 and rHuHFΔ3, respectively, by selective removal of two and three amino acid (aa) residues from the AB loop, located at the *C_3_*–*C_4_* interface [72]. The resulting rHuHFΔ2 and rHuHFΔ3 could disassociate into subunits at pH 3.0 and 4.0, respectively, and small molecules can be accommodated in their cavities by utilizing this superior characteristic. Huard et al. further developed the self-assembled protein architecture by reengineering the *C_2_* interface, which enables the self-assembly of ferritin to be controlled by divalent copper binding [73]. By introducing histidine at i, i + 4 positions of the *C_2_* interface and mutating residues involved in the *C_2_* interface interactions, the variant was obtained that exists stably as a monomer in the absence of Cu(II) and self-assembles to form a cage-like structure through His-Cu(II) coordination under neutral pH conditions. The self-assembly of the ferritin mediated by metal ions provides a new approach for encapsulating guest molecules into the protein cavity.

### 3.2. Expansion of Ferritin Channels by Different Methods for Encapsulation

Apart from the above self-assembly property, ferritin channels are sensitive to chaotrope and temperature. As mentioned before, the high concentration of a chaotropic agent such as urea and guanidine hydrochloride can dissociate ferritin into subunits. To figure out whether chaotropes in a low dose have a similar or different effect on ferritin structures, Yang et al. tried to prepare ferritin-hybrid nanoparticles using 20.0 mM urea [20]. It was found that urea at a low concentration could expand the fourfold channel of phytoferritin, as evidenced by the increased initial iron release rate and decreased α-helix content. Consequently, the guest molecules such as epigallocatechin gallate, chlorogenic acid, or anthocyanin could enter the ferritin cage through the enlarged channels instead of destroying the ferritin structure. After removing urea by dialysis, the channel returns to normal size and guest molecules could be encapsulated in the ferritin nanocage. Moreover, the encapsulation ratio is 17.6% (*w*/*w*), which is comparable to the pH-induced self-assembly method. Moving forward, the 2.0 mM of guanidine hydrochloride (GuHCl) can also be used to prepare ferritin-nutrient co-assemblies by virtue of the sensitivity of ferritin channels to chaotropic agents [74,75]. Its encapsulation mechanism is similar to that induced by urea. Using this method, the encapsulation efficiency of 10.1% was obtained. Compared with the canonical self-assembly approach, low-concentration chaotropic agent-mediated encapsulation can be implemented under benign conditions, which is more suitable for active molecules sensitive to the external environment, while it hardly causes protein damage. However, it is important to note that these methods may trap urea/GuHCl molecules within the ferritin cavity.

On the other hand, temperature has a significant effect on protein structural stability, which may lead to protein misfolding/unfolding and further aggregation [19]. Although the whole ferritin structure is stable against heating to 80 °C for 10 min [8], the channels are reported to be sensitive to temperature changes [9,76]. To investigate whether the thermal treatment can affect the size of ferritin channel, the initial iron release rate (υ_o_) of phytoferritin at different temperatures (20–70 °C) was evaluated [19]. The results show that the initial rate increases in a temperature-dependent manner and reaches the maximum value at 60 °C, which reflects that the fourfold channel of phytoferritin might be expanded to the largest size upon such thermal treatment. Another important finding is that the above-mentioned thermal treatment also reduces the content of α-helix and increases the content of random coils, which is consistent with the iron release result. When the treated temperature decreased to 20 °C, both υ_o_ and α-helical content restored to the original level. Therefore, the guest molecules can be loaded into the ferritin cavity with the encapsulation ratio of 8.08–12.8% through the expansion and recovery of the channels caused by temperature changes. However, many bioactive small molecules are sensitive to temperature, and treatment at 60 °C will lead to degradation and isomerization of these molecules, resulting in a decrease in their activity.

High hydrostatic pressure (HHP) has drawn considerable attention because it could reversibly change protein conformation in the scope of 200–300 MPa by impairing hydrophobic interactions and increasing protein solubility [77]. Previous studies by our research group have shown that HHP treatment had little effect on the primary and secondary structure of soybean seed ferritin (SSF), but greatly changed its tertiary and quaternary structure [78]. Recently, HHP has been used to encapsulate DOX into HuHF instead of using pH or urea to disassemble the protein cage [77]. After varying experimental conditions such as pH, pressure, concentration, additives, and post-treatment protocols, the optimal treatment conditions were obtained with the greatest encapsulation efficiency [79]. On the whole, under optimized conditions, each ferritin could encapsulate 32 DOX molecules, and almost 100% protein could be recovered without any soluble aggregates. However, this method has a complicated system and a long processing time, which may not be suitable for larger size and sensitive cargo molecules.

**Table 1:** Various encapsulation methods for bioactive molecules based on ferritin nanocage. ACP, atmospheric cold plasma; PEF, pulsed electric field; MTS, manothermosonication; EP, extension peptide; HHP, high hydrostatic pressure; DOX, doxorubicin; EGCG, epigallocatechin gallate.

Encapsulation Methods			Guest Molecules	Strengths	Weaknesses	Encapsulation Ratio (*w*/*w*)	Loading Efficiency	References
Self-assembly	Addition of chemicals	HCl/NaOH	curcumin; β-carotene; C3G; metallodrugs	suitable for a variety of molecules	harsh condition; low protein recovery	15–32%	1–3%	[14,17,18,80]
		8 M urea	DOX	suitable for pH-sensitive molecules	protein precipitation; guest molecules waste	-	-	[21]
	Physical methods	ACP/PEF	curcumin/rutin	encapsulation under moderate pH conditions	require sophisticated equipment	12.7–13.7%	-	[65,66]
		MTS	EGCG			25.29%		[68]
	Genetic modification	ΔDE	curcumin/DOX		large pores up to 18 Å at fourfold channel; incomplete protein recovery	-	~1%	[69,70]
		ΔEP	EGCG			11.6%	-	[71]
		Δ^45^DD^46^/Δ^44^RDD^46^	DOX/curcumin		disturb biocompatibility and in vivo performance	-	1.67%	[72]
Channel expansion		20.0 mM urea	EGCG; chlorogenic acid; anthocyanin	little damage to protein; encapsulation without pH adjustment and genetic modification	urea/GuHCl can also be trapped within ferritin cavity; not suitable for larger molecules	17.6%	2.1%	[20]
		2.0 mM GuHCl	rutin			10.1%	-	[75]
		60 °C treatment	rutin/EGCG		may cause guest molecules degradation	8.08/12.8%	-	[19]
		HHP	DOX		long processing time	-	-	[77]

### 3.3. Biomineralization for Preparing Ferritin-Hybrid Nanoparticles

Ferritin has six of the fourfold, eight of the threefold, twelve of the twofold, and twenty-four of the onefold channels, which provide several potential pathways for metal ions and small organic molecules to diffuse into its cavity. As mentioned above, ferritin can deposit free iron ions within the interior cavity in a ferrihydrite form by means of its ferroxidase center. In addition to iron, ferritin can also bind several other metal ions, such as Cu^2+^, Co^2+^, Ni^2+^, Mg^2+^, Mn^2+^, Cd^2+^, Be^2+^, and so on, by incubating the respective metal ions with the empty and intact apoferritin [16]. Taking advantage of this property, several metal/bimetallic nanoparticles [81,82,83,84,85] and metal-containing compounds, such as ^64^CuS [57], ferrocene derivatives [86], Pd(allyl) [87], Rh(nbd) [88], cisplatin [55], ImH[*trans*-RuCl_4_(DMSO)Im] (NAMI A) [89], Ru(II)(η^6^-p-cymene) [90], and [Ru(CO)_3_Cl_2_]_2_ (CORM-2) [91], can also be prepared within the cavity of apoferritin. Briefly, metal salt solution (such as KAuCl_4_) was added to the apoferritin solution at room temperature and neutral pH, and subsequent reduction of the metal ions with reducing agent (such as NaBH_4_), and then dialysis and size-exclusion chromatography were performed to remove unincorporated molecules. The reduction step is omitted when preparing non-zero-valent metal compounds [83]. In particular, there are two strategies for the preparation of bimetallic nanoparticles (NPs). Taking Au/Pd bimetallic NPs as an example, the alloy NPs are prepared by co-reduction of the mixture of Au^3+^ and Pd^2+^ ions in apoferritin; the core–shell NPs are prepared by sequential reduction, in which the Au core is first prepared as a monometallic NP in apoferritin, followed by introduction and reduction of Pd^2+^ ions to form the shell [84]. However, passive diffusion of some metallic compounds into apoferritin turned out to be unfavorable, attributed to the limited pore size of the cage shell. Therefore, many metallodrugs including cisplatin [55], Auoxo3 [92], Auoxo4, Au2phen [93], 4-PF6 [94], Di-Ruthenium-1 [95], [Ru(bpy)_2_dppz]^2+^, [Ru(phen)_2_dppz]^2+^, and [Ru(bpy)_3_]^2+^ [96] have been encapsulated into ferritin cage through biomineralization coupled with pH-induced disassembly and reassembly. The binding efficiency depends on the metal ion, ferritin species, and preparation conditions. For X-ray structures of several ferritin-metallodrug nanocomposites (mainly containing platinum-, ruthenium-, and gold-based anticancer agents), please refer to the recent review article by Merlino and coworkers [80]. Briefly, the data indicate that metal containing fragments from these compounds bind the protein close to diverse sites. Pt and Ru atoms prefer the side chain of His residues, whereas gold atoms bind side chains of both Cys and His, with a preference for Cys [80,94,97,98]. Taken together, apoferritin is a rigid nanoreactor with a fixed volume for biomimetic synthesis.

## 4. Applications of Ferritin-Directed Nanoparticles

### 4.1. Applications in Food Science and Nutrition

Iron deficiency anemia (IDA) is the most common and widespread nutritional disorder in the world, which usually caused by insufficient dietary intakes and low bioavailability of iron [4]. In spite of ferrous sulfate and ferrous gluconate being used as iron supplements, they have some negative consequences and side effects on the human body, such as constipation, diarrhea, and decreased growth [99]. Phytoferritin, especially ferritin from legume seeds, has been considered as a good natural dietary iron source, as it is less susceptible to chelators (phytates and tannins) present in the diet [100]. In legume seeds, about 90% of the total iron is stored within ferritin, and iron atoms could transport across the cell membrane by cell endocytosis in soybean seed [101]. Iron absorption from purified soybean ferritin has been evaluated through the following pathways: healthy, nonanemic women were fed a standardized meal (i.e., bagel, cream cheese, and apple juice) containing 1 µCi ^59^Fe/meal as FeSO_4_ or (extrinsically labeled) as iron-free soybean ferritin reconstituted with the high phosphate characteristic of plant ferritin. The results showed that whole-body iron absorption from soybean ferritin and FeSO_4_ has no significant difference between groups, demonstrating that iron from soybean ferritin is well-absorbed and providing a model for novel, utilizable, plant-based forms of iron for populations with a low iron status [102].

On the other hand, dietary calcium intake is also crucial for human beings, because it is essential for nerve conduction, muscle contraction, mitosis, blood coagulation, and structural support of the skeleton [103]. Although dairy products are a good source of bioavailable calcium, it is not suitable for people in rural areas and strict vegetarians [104]. Additionally, tannic acid and oxalic acid greatly inhibit the absorption of calcium [105], while calcium ions have a negative effect on the absorption of iron and zinc [106,107]. To solve these problems, calcium ions were encapsulated in phytoferritin nanocages, and Ca–protein complexes exhibited two improved features [108]. One is that the calcium ions are protected by the phytoferritin shell from interacting with other dietary factors, such as tannic acid and oxalic acid. The other is that these complexes are absorbed by a new pathway, different from the one for divalent ions; consequently, calcium ions do not interfere with the uptake of iron and zinc. In addition, the calcium–protein complex is suitable for everyone and may help prevent calcium deficiency.

**Figure 5:**
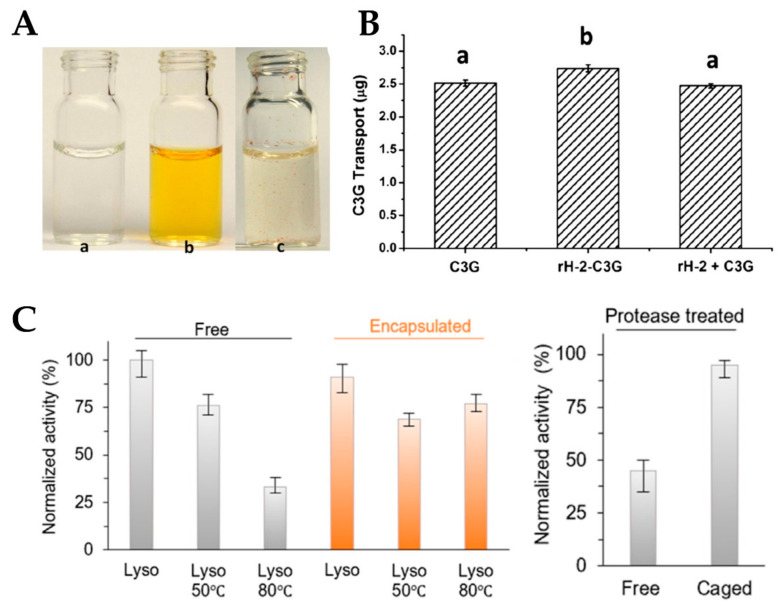
(**A**) Pictures of HuHF alone (a), HuHF-encapsulated curcumin (b), and free curcumin simply mixed with deionized water (c) (Reproduced with permission from [18], *Food Res. Int.*, 2014). (**B**) The amount of the transport of C3G to cell monolayer after 1 h incubation (Reproduced with permission from [14], *Food Res. Int.*, 2014). (**C**) Effect of heat and trypsin on the activity of encapsulated or free lysozyme (Reproduced with permission from [60], *Nano Lett.*, 2019).

Some food bioactive nutrients, such as Cyanidin-3-O-glucoside (C3G) [14], β-carotene [17], curcumin [18], rutin [109], proanthocyanidins (PAs) [110], and lutein [111], have multifarious biological properties including antioxidant, anticancer, and anti-inflammatory activities, but they are extremely sensitive to environmental conditions (pH, light, heat, and oxygen) and prone to degrade/isomerization. Therefore, overcoming the above-mentioned restrictive factors is essential to promote the application of these bioactive components in food and nutrition industries. Recently, ferritin has received more and more interest in encapsulation of bioactive molecules owing to its inherent cavity, high thermal stability, and reversible self-assembly characteristics. After encapsulation, the stability of these nutrients was greatly improved as compared with their corresponding free molecules. Moreover, water solubility can be significantly ameliorated for liposoluble active molecules upon being encapsulated into ferritin nanocage. For example, the curcumin water-solubility increased from 11 ng/mL to 5415 ng/mL (Figure 5A), and the inhibition rate of curcumin degradation can reach 64.99–69.10% from the photothermal stability measurement [18]. The significant improvement in the stability of bioactive nutrients within ferritin nanocages may be attributed to the following three points: (1) ferritin shell may act as a physical barrier to isolate temperature and light, while preventing any pro-oxidants in the water phase from contacting molecules locked in the cavity; (2) ferritin contains cysteine residues and other functional groups that act as effective antioxidants by chelation of transition metals or as free radical scavengers; and (3) ferritin may form molecular complexes with nutrients through hydrogen bond, van der Waals interaction, and hydrophobic forces, which can help protect bioactive molecules from degradation. In addition to stability and water solubility, encapsulation inside the ferritin can also improve the cellular absorption capacity of nutrients. By the Caco-2 cellular absorption analysis of C3G-loaded apo recombinant soybean seed H-2 subunit ferritin (rH-2) nanoparticles, it could be obtained that the absorption and transport efficiency of C3G encapsulated in ferritin shell is higher than that of free C3G molecules (Figure 5B). As reported, the pathways corresponding to ferritin and anthocyanins uptake by Caco-2 cells are related to AP2-dependent endocytosis and to GLUT2 transporter, respectively. The results showed that C3G adheres to the apical brush border microvilli more efficiently than apoferritin. This may explain the difference in absorption and transport efficiency between free C3G and rH-2-C3G [14]. Thus, ferritin as the nanocarrier can not only improve the water solubility and stability of nutrients, but also facilitate their absorption and transport in cells.

Lysozyme, also known as muramidase or N-acetylmuramic hydrolase, is present in the organs and secretions of human, vertebrates, bacteria, and even plants [112]. Among them, hen egg-white lysozyme (HEWL), as a result of abundant resources and antimicrobial ability, has attracted a great deal of attention, and has been used as a food preservative to extend the shelf life of products and to enhance the safety of foods [113]. Until now, HEWL has been the only lysozyme allowed for use in the food industry [114]. However, lysozyme can be degraded by trypsin, and its activity is sensitive to temperature changes. Recently, lysozyme has been encapsulated into the TmFtn by taking advantage of salt-mediated assembly properties of the protein (Figure 5C) [60]. Surprisingly, the encapsulated lysozyme showed very high activity at 80 °C, while the free enzyme showed significantly decreased activity. This can be expected because the denaturation temperature of the free enzyme is about 75 °C, and such a thermo-protective effect has been reported in the literature, such as encapsulating the artificial (retro-)aldolase RA95.5-8F in AfFtn [61]. Furthermore, compared with the encapsulated enzyme, free lysozyme was more susceptible to digestion in the presence of trypsin, resulting in reduced activity.

Mercury is a highly toxic heavy metal, which can be bio-accumulated through the food-chains, especially in seafood and marine fish. In general, organic mercury is more toxic than inorganic mercury, and in organic mercury, methyl mercury (MeHg^+^) is the most harmful to the human body [115]. Because of its small molecular weight and strong fat solubility, methyl mercury can easily pass through the blood–brain barrier, and thus exhibits strong neurotoxicity. Therefore, the monitoring of mercury concentrations in foodstuffs and drinking water is important to ensure compliance with food safety regulations and consequent consumer protection. Au nanoclusters (NCs) have been widely utilized in chemical sensing and biological imaging thanks to their intrinsic fluorescent properties, low toxicity, easy preparation, and excellent chemical stability. Further, they also can be used for Hg^2+^ detection in aqueous solution based on the specific interaction between mercury and Au NCs [116]. Even though several Au NCs sensors have been synthesized for Hg^2+^ detection thus far, their applications are often limited by poor water solubility, low sensitivity and selectivity, and fluorescent interference from other components [117]. Recently, by utilizing the non-native 16-mer lenticular protein (7A) as bio-template, our group fabricated a novel Au NCs, which can detect Hg^2+^ and MeHg^+^ in vitro and in vivo, respectively, with high selectivity. The prepared 7A-Au NCs have the ability to be a bioimaging detection probe for MeHg^+^ in the brain of living mice [118]. Thus, it appears that the nanoparticles prepared with ferritin as a template hold great promise for the detection of heavy metal ions in the field of food safety.

### 4.2. Applications in Medicine and Diagnostics

**Figure 6:**
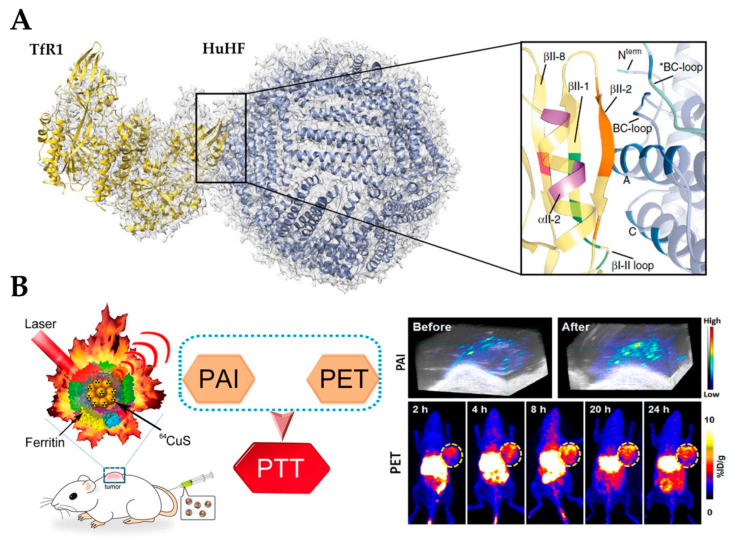
(**A**) Atomic model of the complex of TfR1 (yellow) and HuHF (light blue) fitted in the cryo-EM map Reproduced with permission from [120], *Nat. Commun.*, 2019). (**B**) ^64^CuS–Fn NCs as clinically translatable cancer theranostics for positron emission tomography and photoacoustic dual-modality imaging (PET/PAI) dual-modal imaging guided photothermal therapy (PTT) (Reproduced with permission from [57], *ACS Nano*, 2016). NC, nanocluster.

Cancer is the leading cause of death worldwide. The modern therapeutic strategy for cancer is to selectively remove the tumor before it evolves into its superior stages and finally metastasizes. On the basis of the current situation, it is important to develop novel drug formulations to enhance the efficacy of cancer cells, while reducing the toxicity to healthy cells. On the other hand, more sensitive diagnostic tools are also necessary to improve the screening accuracy and detection rate of malignant tumors, and reduce surgery or drug treatment [119]. Compared with other drug delivery systems like liposomes and mesoporous silica, ferritin is more desirable because of its stability, biocompatibility, and biodegradability. In addition, it is worth noting that ferritin has site-specific targeting potential, as it can be recognized and internalized by ferritin-binding receptors, such as the transferrin receptor 1 (TfR1) for H-ferritin (Figure 6A) and scavenger receptor class A member 5 (SCARA5) for L-ferritin, which are over-expressed in a variety of tumor cells, especially in liver cancer cells [120,121]. Therefore, ferritin nanocage has been developed into a bio-template for the encapsulation and delivery of anticancer drugs and tumor imaging agents, and applied in chemotherapy, photothermal therapy, and vivo imaging.

Until now, several metallodrugs including cisplatin, carboplatin, oxaliplatin [55,122,123], Auoxo3 [92], Auoxo4, Au2phen [93], 4-PF6 [94], NAMI A [89], Ru(II)(η^6^-p-cymene) [90], Di-Ruthenium-1[95], [Ru(bpy)_2_dppz]^2+^, [Ru(phen)_2_dppz]^2+^, and [Ru(bpy)_3_]^2+^ [96], as well as non-metallic drugs including DOX [21], curcumin [124], and cytochrome C [62], have been encapsulated in ferritin cavity to improve their bioavailability, tumor-selectivity, and cellular permeability and reduce the toxicity to normal cells. Guo and co-workers first proposed the cisplatin encapsulation and explored the cell absorption of ferritin-cisplatin nanoparticles (NPs) and their applications in tumor therapy. The encapsulation was achieved through manipulating the pH-based disassembly–reassembly process of apoferritin in saturated drug solution with pH 2.0 and 7.4, respectively. The cell experiments showed that ferritin-cisplatin NPs could inhibit the growth of tumor cells slowly and continuously, and enhance the cell absorption of cisplatin [55,123]. Similar to cisplatin, alternative metal compounds based on Ru and Au have been prepared and evaluated for their cytotoxic activity in recent years, mostly by Merlino and co-workers [89,92,93,94,95]. The adducts of these compounds with ferritin are less toxic than free drugs and are moderately selective towards cancer cells compared with non-malignant cells [80]. Moving forwards, HuHF-DOX NPs was prepared through a disassembly–reassembly process driven by 8 M urea. From the fluorescence microscopy observation of HT-29 tumor cells, it was found that HuHF-DOX specifically bound and internalized into tumor cells by interaction with TfR1, and released Dox in the lysosomes, which leads to the intracellular drug release and tumor cell apoptosis. More importantly, compared with free Dox, the drug concentration of HuHF-Dox in the tumor is more than 10 times higher, while the drug dose in healthy organs is significantly reduced [21]. Recently, humanized Archaeoglobus ferritin (HumFt) was constructed by grafting the BC loop from HuHF onto AfFtn, which combines the versatility in assembly and cargo incorporation properties of AfFtn with binding capabilities and cellular uptake properties of HuHF. By exploiting the unique properties of HumFt to encapsulate and deliver bioactive full-length cytochrome C to tumor cells, the dual fluorescent labeling results demonstrated that effective liberation of cytochrome C within the cytosolic environment can induce cancer cell apoptosis [62]. In short, tumor targeting potentiality, relative high loading capacity, and good water solubility make apoferritin a promising carrier for anticancer drugs.

Besides drugs, some metal nanoparticles and dye molecules have been loaded into ferritin nanocages for magnetic resonance imaging (MRI) and optical imaging, which play vital roles in early recognition, diagnosis, monitoring, and prognosis of cancer. Currently, gadolinium-based contrast agents lack specificity for cancer cells and the spatial resolution may not be sufficient to detect occult cancer microdeposits. To overcome these limitations, single-crystal cores of magnetite were synthesized inside the ferritin cage as the MRI contrast agent. This complex exhibited extremely high transversal relaxivity (r_2_) of 224 mM^−1^ s^−1^ and TfR1-dependent MRI signal [125]. Using the hollow cavity of ferritin as a nanoreactor, many other MRI-detectable nanoparticles have been prepared [126,127,128], such as ferritin-gadolinium nanoparticles, whose longitudinal and transverse relaxation is 10 to 70 times higher than that of commercially available Gd chelates. Light, typically a wavelength-selected laser, as an exogenous stimulus with the advantage of spatiotemporal selectivity has been extensively employed for photothermal, photodynamic, and/or photo-triggered chemo/gene therapy. Recently, ferritin-^64^CuS NPs have been synthesized by the biomimetic synthesis method for simultaneous positron emission tomography (PET) and photoacoustic dual-modality imaging (PET/PAI) guided photothermal therapy (PTT) (Figure 6B). The nanoparticles have good biocompatibility, distinct near-infrared (NIR) absorbance, strong photoacoustic contrast, high photothermal conversion efficiency, and could be monitored by PET and PAI. Notably, the tumor could be completely eliminated by iv injection of Ferritin-^64^CuS NPs with a low laser irradiation dose [57]. Altogether, the reported literature indicates that ferritin nanocage represents a promising nano-vehicle for tumor imaging and photothermal therapy.

## 5. Challenges of Ferritin as a Nanocarrier

As a nanocarrier for the encapsulation and delivery of nutrients and drugs, the digestive stability of ferritin in gastrointestinal tract has received wide attention from researchers. If ferritin cannot survive through gastrointestinal digestion, the prepared nanoparticles will make little contribution to human nutrition and or disease treatment through any absorption pathway [25]. However, a recent report has found that ferritin is not stable in the stomach, and most ferritin molecules can be degraded by pepsin at pH 2.0 in this region [129]. It should be clarified that, although the adult gastric pH is considered to be 2.0, the pH is actually about 4.0 within 3 h after a meal, which is the reason that most simulated gastric fluid digestion is conducted at pH 4.0 [130,131]. Previous studies have discovered that the interaction of ferritin with EGCG, Pas, and gallic acid molecules can effectively inhibit the degradation of ferritin by pepsin at pH 4.0 [132,133]. This may be due to the combination of polyphenols with the protein cage through van der Waals interactions or hydrogen bonds, thereby improving the stability of the protein. Not only the weak forces, but also the covalent effect of ferritin and chitosan has been used to improve the stability of the protein. Recently, the recombinant soy bean seed H-2 type ferritin was glycosylated by chitosan to fabricate a ferritin–chitosan covalent composite that was more resistant to pepsin and trypsin digestion than ferritin alone [134]. For plant ferritin, the composition of subunits is also an important factor affecting protein stability, as H-2 is more resistant toward proteolysis compared with H-1. It has been reported that ferritin with a higher H-2/H-1 ratio exhibits a stronger digestive stability at pH 4.0. Moreover, the effect of heat treatment and skim milk on ferritin stability in vivo has been evaluated. By detecting HSF in the gastrointestinal tract of suckling rat pups in the presence of skim milk, it was found that the skim milk may have a protective effect on HSF during digestion, but heat treatment may lead to the decline of its digestive stability. The protective effect may be attributed to the interaction between HSF and molecules in skim milk, which can stabilize the structure of ferritin, but the specific mechanism is still unknown [135].

Inevitably, the degradation of ferritin affects the release rate of the encapsulated molecules to a large extent. Consistent with the above conclusions, recent studies have found that certain food matrices (such as EGCG, PA, and milk) can inhibit the release of lutein, which is encapsulated in the ferritin cavity. However, pectin can promote the release of lutein from the shell, which indicated that different types of food matrices have different effects on the release of bioactive molecules [111]. A similar inhibitory effect can also be obtained in the release of rutin, which may be due to the interaction of some food matrices with ferritin, which reduces the sensitivity of the protein to enzymes and improves protein stability, resulting in the slowing release of nutrients [56]. Recently, the transport of free epigallocatechin (EGC), EGC-loaded apo-red bean (*adzuki*) ferritin (ER), and ER-chitosan nanoparticles (ERCs) across the Caco-2 cell and TfR1 silent Caco-2 cell monolayer has been monitored to explore whether food matrices can influence the cell absorption efficiency of encapsulated molecules [136]. The results showed that the absorption of EGC in ER and ERCs (the molecular weights of chitosan is 980/4600 Da) across the Caco-2 cells was enhanced by the TfR-1-mediated pathway. Furthermore, despite this, in the TfR1 silent Caco-2 cell monolayer model, the transport efficiency of EGC in ERCs980 was significantly higher than that in ER, indicating that chitosan with a lower molecular weight was beneficial to the absorption of EGC. The positive effect of chitosan on EGC transport may be due to its special transcellular and paracellular pathways, that is, it can not only penetrate across the small intestinal epithelium through the paracellular or transcellular pathway, but also can open tight junctions between epithelial cells to promote paracellular transport [137]. However, the detailed mechanism and the cytotoxicity analysis need further investigation.

## 6. Conclusions and Perspectives

Although ferritins are widely distributed in plants, animals, and microorganisms, there are still a few differences between their structures and properties. This review focuses on the preparation of ferritin-hybrid nanoparticles and their applications in the food, nutrition, and medicine industries. Not only are the reversible disassembly–reassembly characteristics emphasized in the fabrication and delivery guest molecules, but also several novel strategies suitable for pH-sensitive molecules are introduced. These new approaches can achieve encapsulation of guest molecules into the ferritin cavity under neutral conditions by the addition of chemical agents or physics-assisted. Ferritin nanocarriers can improve the water solubility, stability, cell absorption efficiency, as well as targeted delivery of guest molecules. Moreover, some food matrices can be modified on the outer surface of the protein to inhibit the degradation of ferritin in the gastrointestinal tract and promote the bioavailability of guest molecules. Even though significant progress has been achieved in the preparation and application of nanoparticles based on ferritin nanocarrier, there are still some bottlenecks that need to be addressed. First, the encapsulation efficiency and loading capacity need to be further improved. One way to achieve this goal is to apply non-native ferritin cages of different sizes or shapes obtained through genetic or chemical modification. Second, it is necessary to realize multi-component encapsulation to cope with multi-drug resistance. Third, most of the current research focuses on preparation methods and medical applications, and more attention should be paid to the application of ferritin-hybrid nanoparticles in food nutrition, detection, and screening. Finally, the ferritin stability and the cellular uptake efficiency of guest molecules need to be further improved, and the mechanism and effects of ferritin surface modification need to be explored in depth.
